# Intraspecific variation of trace elements in the kelp gull (*Larus dominicanus*): influence of age, sex and location

**DOI:** 10.1016/j.heliyon.2021.e05994

**Published:** 2021-01-18

**Authors:** Jorge Henrique Pedrobom, Amauri A. Menegário, Hendryk Gemeiner, Everton Tiago Sulato, Lucas Pellegrini Elias, Patrícia Pereira Serafini, Claudinei José Rodrigues, André S. Barreto, Marcus Antônio Gonçalves de Araújo Júnior

**Affiliations:** a Environmental Studies Center (CEA), São Paulo State University (UNESP), Avenida 24-A, 1515, CEP 13506-900, Rio Claro, SP, Brazil; b Chico Mendes Institute for Biodiversity Conservation – ICMBio, Rodovia Jornalista Maurício Sirotski Sobrinho, km 2, CEP 88053-700, Florianópolis, SC, Brazil; c Biodiversity and Geoprocessing Informatics Laboratory, University of Vale do Itajaí (UNIVALI), Rua Uruguai, 458, CEP 88302-901, Itajaí, SC, Brazil; d Research and Development Center Leopoldo Américo Miguez de Mello – CENPES, PETROBRAS - Petróleo Brasileiro S.A, Avenida Horácio Macedo, 950, CEP 21941-915, Rio de Janeiro, RJ, Brazil

**Keywords:** Intraspecific variation, Trace elements, Hepatic tissue, *Larus dominicanus*

## Abstract

Hepatic tissue of *Larus dominicanus* sampled on the coastline of the state of Santa Catarina in Brazil between October 2016 and May 2018 was used to evaluate intraspecific trends and spatial distribution of essential trace elements (Mn, Co, Cu, Zn, Mo and Cr) and non-essential trace elements (As, Pb, Cd, Hg, Ba and V). Principal Component Analysis (PCA) indicated differences in the bioaccumulation of trace elements between female adults and male adults, differences to sex and age were indicated by Kruskal-Wallis test. Heat maps suggest hot spots in locals with high concentration of trace elements in liver of *Larus dominicanus*. In general, the concentration of trace elements were comparable with values reported in other studies carried out for this species in South America and other regions of the world. The heat maps showed to be a promising tool to identify influences of the locality on bioaccumulation of trace elements in *Larus dominicanus*.

## 1 Introduction

Marine environments are vulnerable to contamination by pollutants originated by anthropogenic activities like the burning of fossil fuels, oil spills, production of chemicals as well as mining and agricultural activities in coastal areas (Halpern et al., 2008). As oceans are the ultimate sink of trace metals, the contamination of coastal areas by metals represents a threat for marine species (García-Tarrasón et al., 2013). Organisms on top of the food chain as seabirds are especially vulnerable to trace metal contamination due to biomagnification along the food web (Burger and Gochfeld 2004). The accumulation of elevated concentrations of trace metals by birds is extremely harmful for the individuals and leads to a wide range of acute and health effects (Rocque and Winker 2004).

The Santos Basin Beach Monitoring Project (Projeto de Monitoramento de Praias da Bacia de Santos PMP-BS) is coordinated by the principal Brazilian oil company, Petróleo Brasileiro S.A – PETROBRAS. This project is one of the monitoring programs required by the Brazilian Federal Environmental Agency (IBAMA) for the environmental licensing of oil and natural gas production and their transport by Petrobras at the Pré-Sal Province (25⁰05′S 42⁰35′W to 25⁰55′S 43⁰34′W). The aims of PMP-BS are to evaluate the influence of oil exploration activities on seabirds, turtles and marine mammals at the Pré-Sal Province of the Santos Basin. The program project involves the sampling of individuals found stranded during beach surveys at a corridor of 1500 km at the coastline of Brazil (Godoy et al., 2020).

The Kelp gull (*Larus dominicanus*) is a bird species monitored by the PMP-BS. This species can be found along more than 2000 km at the coastlines in South America (Branco et al., 2009). The feeding habits of *Larus dominicanus* are based mainly on fish and invertebrates, but the species exhibits flexibly habits and can easily adapt to urban environments, feeding on garbage and food debris as well as fishery discards (Miotto et al., 2016). In this sense, improving the knowledge about *Larus dominicanus* can be important to understand the bioaccumulation and toxicity of metals by birds in coastal areas (Parrish and Zador, 2003, Vieira and Serafini, 2016).

Some works have shown the accumulation of different trace metals in liver samples of *Larus dominicanus* in the southern hemisphere and other parts of the world. Vermeer and Castilla. (1991) observed a high variability of Cd and Cu concentrations in the livers of *Larus dominicanus* specimens collected downstream a mining activity area in northern Chile. Numata et al. (2008) compared the concentrations of As, Cd, Cu, Hg, Pb and Zn determined in the livers of *Larus dominicanus* and *Tadorna variegata* and the activity of biomarkers of oxidative stress. The authors reported a relationship between the liver concentrations of Hg and Pb and the biomarkers response. Additionally, the low body weight observed in individuals where contaminant concentration was higher was consistent with the oxidative stress response, indicating the high energetic demand of detoxifying processes. Cortés and Luna-Jorquera 2011 compared the Cd and Cu concentrations in the livers of adult and juvenile individuals collected from a coastal area of Chile. The significant differences in Cd concentrations between juveniles and adults suggested that Cd is bioaccumulated by the birds, while Cu is effectively regulated.

The sampling procedures involved in using liver to study the bioaccumulation in birds are often performed using invasive sampling techniques based on euthanasia of individuals (Burger and Gochfeld 2000). In this regard, the sampling of feathers is not lethal for the birds and is commonly used to monitor environmental concentrations of trace elements (Barbieri et al., 2010; Ebert et al., 2020). However, environmental monitoring of metals based on concentrations in feathers is limited since feathers are substituted during the lifetime of birds leading to underestimation of the real biological effects and bioaccumulation of trace elements (Monteiro and Furness 1955; Pereira Filho et al., 2003; Honda et al., 1985).

The sampling of birds in coastal areas for environmental monitoring of contaminants is hindered by the high mobility of the seabirds combined with the low density of the populations (Clatterbuck et al., 2018). In addition, the use of invasive approaches for sampling the birds is also ethically questionable (Lodenius and Solonen 2013). An ethically justifiable alternative is the sampling of dead individuals found stranded on beaches which enables the employment of standardized protocols and avoids invasive sampling (Parrish et al., 2007).

Moura et al. (2018) evaluated interspecific differences on the accumulation of essential and non-essential trace metals measured in liver of four species of seabirds (including *Larus dominicanus*) sampling dead individuals found stranded in beaches along the Brazilian coast. The differences in feeding behaviour influenced the concentration of the trace metals determined in the hepatic and muscular tissues of sympatric birds. Furthermore, a relationship between the levels of Se and Hg indicated that the formation of a Se–Hg complex is involved in the detoxification process of methyl mercury in seabirds.

In the present work, the concentrations of essential (Mn, Co, Cu, Zn, Mo and Cr) and non-essential (As, Pb, Cd, Hg, Ba and V) trace metals in hepatic tissue of *Larus dominicanus* specimes found stranded along the coast of Santa Catarina state, South Brazil, between October 2016 and May 2018, were determined. The data generated was used to imply spatial distribution patterns of the environmental concentrations of trace metals as well as intraspecific trends related by age and sex in the bioaccumulation of trace metals by populations of *Larus dominicanus.*

## 2 Materials and methods

### 2.1 Study area

The state of Santa Catarina is located in the southern region of Brazil and has a 539 km long coast on the Atlantic Ocean. Different types of human activities affect each mesoregion of the state. In the north of the state of Santa Catarina, the construction of a landfill in the Linguado channel in the estuary of São Francisco do Sul caused the hydrodynamic interruption in Babitonga Bay. The intense silting of the channel caused the sedimentation of effluents containing trace elements from industry (Engel et al., 2017). Recent studies have shown high concentrations of trace elements in fish sampled in this region, showing the bioavailability of these trace elements in the environment (Demori, 2008).

In the Itajaí Valley, the main changes in the environment are associated with the Itajaí-Açu river estuary. The estuary receives a contribution from a drainage basin of approximately 15.500 km^2^ (25% of the state area). The cities of Blumenau and Brusque are largely industrialized. The Itajaí-Açu river estuary is economically important, since it is located in the largest port in the state of Santa Catarina (Itajaí port) (Filho et al., 2006). Along the estuary, dozens of industries are installed linked to the improvement of local fishing. The residue of these activities is generally released in the estuary without previous treatment (Frena et al., 2016).

The main anthropic activity in the region of Florianópolis is associated with disorderly occupation of the coastal environment (30% of the state's population lives in Florianópolis). In addition, the region is surrounded by urban beaches where solid wastes are disposed constantly into the environment, which can substantially increase the exposure of trace metals (Filho, 2003).

Finally, the southern region of Santa Catarina state has among its main human activities the exploration of large reserves of coal located in the Rio Bonito region. The coal of the region contains noteworthy amounts of metal complexes as Al_2_O_3_ (14.5% m m^−1^), Fe_2_O_3_ (6.9% m m^−1^), Co (15.7 mg kg^−1^), Cr (54.4 mg kg^−1^), Cu (30 mg kg^−1^), Mn (150.4 mg kg^−1^) and Pb (58 mg kg^−1^) (Kalkreuth et al., 2010).

### 2.2 Sampling of *Larus dominicanus* specimen

**Figure 1:**
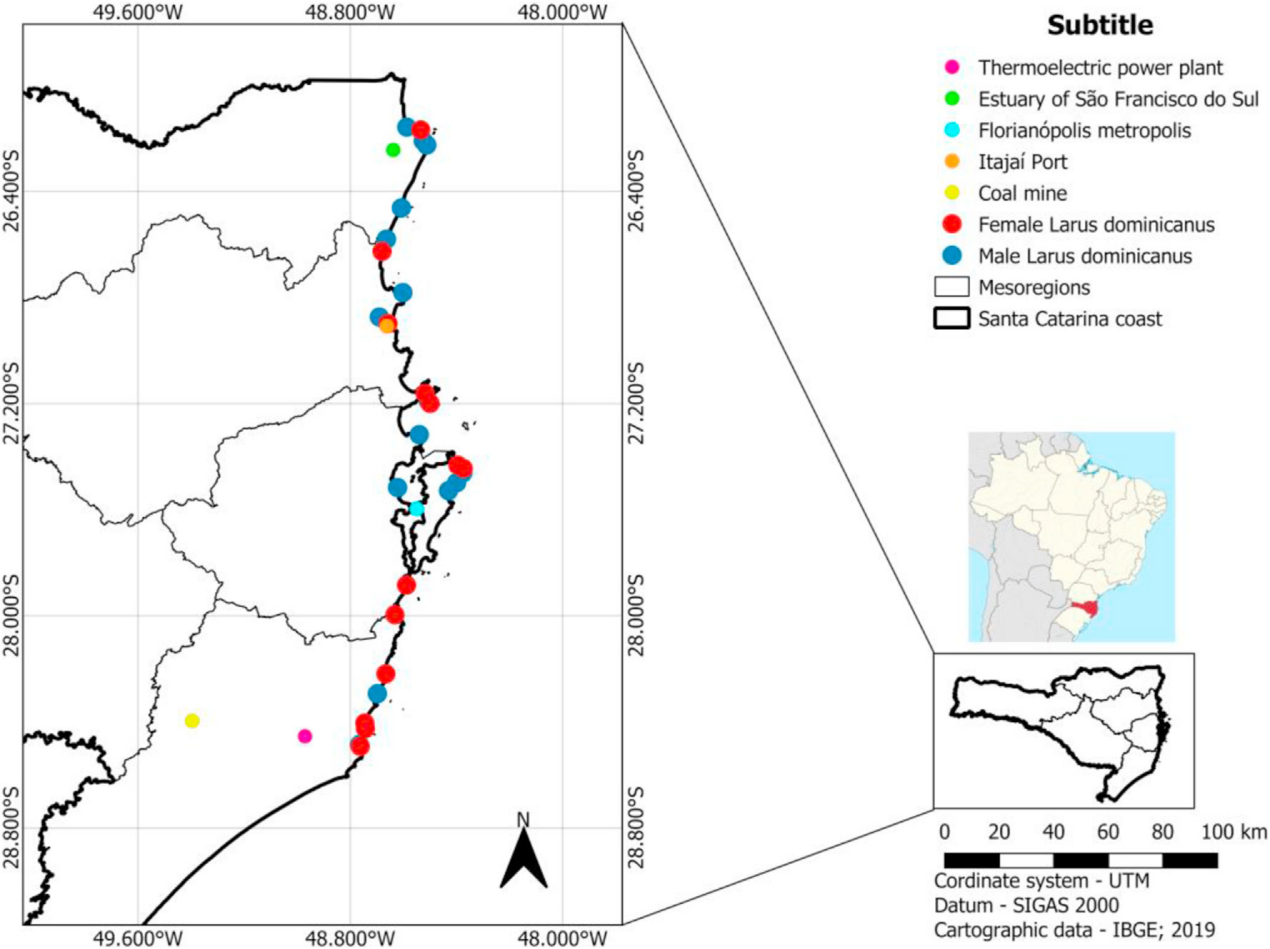
*Larus dominicanus* found in Santa Cataria State.

The study area covers a coastline of around 264 km of Santa Catarina state between the cities of Laguna and Itapoá (Figure 1). The 30 individuals of *Larus dominicanus* sampled for this study were found dead on the beaches during the regular monitoring of the PMP-BS project. The decay status of each individual was defined during necropsy, and only fresh carcasses in appropriate conditions were collected and taken to laboratory for further analysis (Geraci and Lounsbury, 1993).

At the laboratory, sex and age (juvenile or adult based on the plumage) of individuals were defined. Samples of liver tissue were collected during the necropsy using a ceramic knife. Liver samples were stored in polypropylene flasks at -80 °C.

### 2.3 Trace metal determination

The liver samples were thawed and manually homogenized using polypropylene mortar and pestle. The samples were then separated in two portions and directed to the pre-treatment process for the determination of As, Ba, Cd, Cr, Cu, Pb, Mn, Mo, V and Zn by Inductively Coupled Plasma Mass Spectrometry (ICP-MS X Series II, Thermo Scientific, Germany) and determination of Hg by Cold Vapor Atomic Fluorescence Spectrometry (CV-AFS) (PS Analytical model Millennium Merlin, UK). The concentrations of all trace elements are expressed in dry weight (mg kg^−1^ d.w.).

#### 2.3.1 As, Ba, Cd, Cr, Cu, Pb, Mn, Mo, V, and Zn

An amount of 5 g of each liver sample was lyophilized (Alpha 1–2 LDplus-Christ) at -40 °C and 0.11 mbar for 72 h. The samples were weighed before and after the lyophilization procedure to estimate average water content in liver samples. After lyophilization, the samples were conducted to the acid digestion process using a microwave system (Speedwave Four, Berghof, Germany). A mass of 0.175 g of dry liver sample was added to 5 mL of HNO_3_ sub-boiling acid and heated to 170 °C for 5 min, after this time, the samples were heated to 200 °C for 15 min. The digested liver samples were then transferred to polypropylene flasks and were appropriately diluted with ultrapure water (Milli-Q, Millipore, EUA). Finally, the samples were conducted to the analysis by ICP-MS. The accuracy of the procedure was validated using reference material Lobster Hepatopancreas TORT-3 (National Research Council Canada).

The ICP-MS was equipped with a collision cell (CCT) containing He gas, a nebulizer type *Miramist* and a refrigerated nebulization chamber. The ICP-MS was operated with the following settings: plasma power = 1300 W, nebulizer gas flow = 0.8 L min^−1^, coolant gas flow = 13 L min^−1^, auxiliary gas flow = 0.7 L min ^−1^ and CCT gas flow = 5 mL min^−1^. The used quantification mode was peek jump with 100 sweeps, a dwell time of 10 ms of each trace metal and 3 replicates per run. The concentration determination of As, Cr, Cu, Mn, Cr, Zn and V were performed using a collision cell (CCT) mode. During the analysis, a solution containing of ^45^Sc, ^89^Y, ^103^Rh, ^115^In, ^159^Tb, ^169^Ho and ^209^Bi was introduced *online* and used as an internal standard.

#### 2.3.2 Hg

The portion of liver samples directed to Hg analysis was directly digested without lyophilization since there can be Hg losses during the lyophilization process. A mass of 1 g of liver sample was added to 2 mL of HNO_3_ sub-boiling acid and 6 mL of HCl sub-boiling acid. The solution was kept in this “pre-digestion” procedure for 12 h. After that step, the samples were heated to 170 °C for 5 min, and subsequently samples were heated to 200 °C for 15 min. The digested samples were then transferred to polypropylene flasks and dilutions were appropriately performed.

As requested for the analysis of Hg by CV-AFS, a volume of 1.5 ml of KBr (1.19 % m v^−1^) and KBrO_3_ (0.28 % m v^−1^) solution and 0.75 ml HCl (m v^−1^) sub-boiling acid were added to the sample. After 30 min, the excess of KBr and KBrO_3_ was removed with 0.002 ml of hydroxylamine solution (12 % m v^−1^). The reduction of Hg^+ 2^ to Hg^0^ in the standard solution and samples was performed using SnCl_2_ (stannous chloride) at 2% (m V^−1^) dissolved in 10% (V V ^−1^) of hydrochloric acid. The reducing agent (SnCl_2_) was added online along with analytical standards and samples. The CV-AFS is equipped with an automatic switching valve, the measurement was realized in emission mode with a signal gain of 100 and signal measurement mode of peak height. The accuracy of the procedure was validated using reference material Lobster Hepatopancreas TORT-3 (National Research Council Canada).

### 2.4 Statistical analysis

A multivariate principal component analysis (PCA) was used only regarding adult specimens of *Larus dominicanus* to identify differences in the bioaccumulation of trace elements related to sex. The PCA results were normalized using the mean and standard deviations of the concentrations of trace metals. PCA was performed by PAST 3.0 software.

The multivariate statistical test (Kruskal-Wallis) was performed by Originlab software (Version 2018). Kruskal-Wallis tests assuming 95 % of significance level were used to identify differences in the bioaccumulation of trace elements in different classes (sex and age).

The spatial statistical analysis was performed from upload open files of maps obtained from the Brazilian Institute of Geography and Statistics (IBGE) and QGIS software (Version 3.14.1). Kernel maps were used to estimate the geographical distribution of trace metal concentrations and identify areas with high possibility of concentration of trace elements. The concentration value of each trace element was correlated with geospatial data of the respective sampling point. The quadratic Kernel function was applied using a radius the 11.1 km to generate the heat maps. The resolution used in the Kernel maps was 2535 pixel by line and 592 pixel by column.

## 3 Results

Recoveries between 80 and 120 % of TORT 3 were observed (Table 1). Limits of detection (LD) and Limits of Quantification (LQ) were substantially lower than the concentration values obtained for the study samples (Table 1).

**Table 1:** Trace metal concentrations determined in TORT 3, limits of detection (LD) and limits of quantification (LQ).

Trace elements	TORT-3 [R ± DPR %]	LD [mg kg^−1^ d.w.]	LQ [mg kg^−1^ d.w.]
As	101 ± 7	0.03	0.1
Cd	95 ± 5	0.02	0.04
Cr	91 ± 12	0.1	0.2
Cu	77 ± 5	0.1	0.2
Pb	100 ± 8	0.01	0.05
Mn	85 ± 7	0,1	0.2
Mo	82 ± 4	0.1	0.2
Zn	99 ± 5	5	15
Ni	90 ± 5	0.1	0.2
Ba	-	0.1	0.2
V	90 ± 5	0.02	0.07
Hg	87 ± 8	0.04	0.1

### 3.1 Trace metal variation - sex and age

The variability of trace metal concentrations was noteworthy when comparing the different age classes. Higher mean concentrations of As (5.9 mg kg^-1^ d.w.), Cd (0.5 mg kg^-1^ d.w.), Cu (15 mg kg^-1^ d.w.), Pb (1.2 mg kg^-1^ d.w.), Mn (13 mg kg^-1^ d.w.), Zn (254 mg kg^-1^ d.w.) and Hg (4.8 mg kg^-1^ d.w.) were observed in female *Larus dominicanus* (Table 2). Male *Larus dominicanus* presented higher mean concentrations of Cr (0.1 mg kg^-1^ d.w.), Ba (0.1 mg kg^-1^ d.w.) and V (0.8 mg kg^-1^ d.w.) (Table 2). The relative standard deviation (RSD %) of As, Pb and V were greater than the mean concentration.

**Table 2:** Concentrations of trace metals (mg kg^-1^ d.w.) in liver of *Larus dominicanus*.

	Sex		Mean ± sd	Maximum	Minimum
As	M	J (n = 6)	2.6 ± 2	7	0.4
		A (n = 8)	2.2 ± 2		
	F	J (n = 2)	5.9 ± 1	12	0.2
		A (n = 14)	2.6 ± 3		
		Total (n = 30)	2.7 ± 3		
Cd	M	J (n = 6)	0.1 ± 0.1	0.8	0.1
		A (n = 8)	0.4 ± 0.2		
	F	J (n = 2)	0.2 ± 0.1	1.2	0.1
		A (n = 14)	0.5 ± 0.3		
		Total (n = 30)	0.4 ± 0.3		
Cr	M	J (n = 6)	< LD	0.30	<LD
		A (n = 8)	0.1 ± 0.1		
	F	J (n = 2)	< LD	< LD	< LD
		A (n = 14)	< LD		
		Total (n = 30)	< LD		
Cu	M	J (n = 6)	13 ± 3	18	9
		A (n = 8)	14 ± 2		
	F	J (n = 2)	9 ± 0.3	26	9
		A (n = 14)	15 ± 4		
		Total (n = 30)	14 ± 3		
Pb	M	J (n = 6)	0.06 ± 0.03	0.5	0.02
		A (n = 8)	0.14 ± 0.15		
	F	J (n = 2)	0.06 ± 0.03	16	< LD
		A (n = 14)	1.20 ± 4		
		Total (n = 30)	0.6 ± 3		
Mn	M	J (n = 6)	10 ± 3	15	6
		A (n = 8)	12 ± 2		
	F	J (n = 2)	13 ± 1	15	4
		A (n = 14)	11 ± 3		
		Total (n = 30)	11 ± 3		
Mo	M	J (n = 6)	1.8 ± 0.4	2.7	1.4
		A (n = 8)	2.0 ± 0.4		
	F	J (n = 2)	1.8 ± 0.5	2.8	0.9
		A (n = 14)	2.0 ± 0.5		
		Total (n = 30)	1.9 ± 0.4		
Zn	M	J (n = 6)	177 ± 110	303	71
		A (n = 8)	114 ± 77		
	F	J (n = 2)	254 ± 183	384	36
		A (n = 14)	107 ± 52		
		Total (n = 30)	133 ± 88		
Ni	M	J (n = 6)	< LD	0.1	< LD
		A (n = 8)	< LD		
	F	J (n = 2)	< LD	< LD	< LD
		A (n = 14)	< LD		
		Total (n = 30)	< LD		
Ba	M	J (n = 6)	0.1 ± 0.1	0.2	< LD
		A (n = 8)	< LD		
	F	J (n = 2)	< LD	0.1	< LD
		A (n = 14)	< LD		
		Total (n = 30)	< LD		
V	M	J (n = 6)	0.3 ± 0.2	4.6	0.1
		A (n = 8)	0.8 ± 1.6		
	F	J (n = 2)	0.2 ± 0.3	0.5	0.01
		A (n = 14)	0.2 ± 0.1		
		Total (n = 30)	0.4 ± 0.8		
Hg	M	J (n = 6)	3.6 ± 2	6.2	0.4
		A (n = 8)	2.6 ± 2		
	F	J (n = 2)	4.8 ± 0.1	6.1	0.8
		A (n = 14)	2.9 ± 2		
		Total (n = 30)	3.1 ± 2		

M = male, F = female, J = juvenile, A = adult.

**Figure 2:**
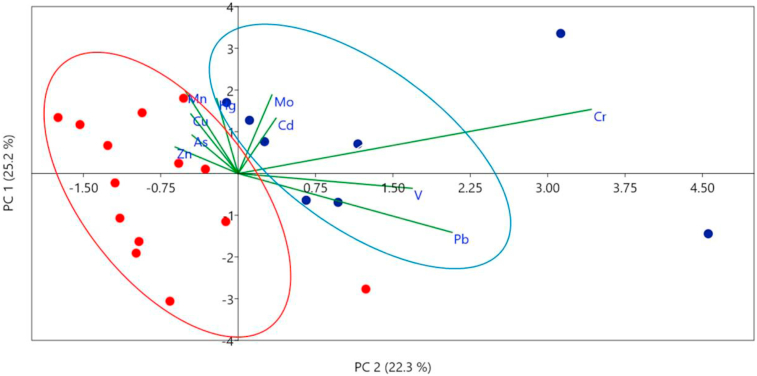
Principal Component Analysis (PCA) of trace element concentrations in liver of *Larus dominicanus.* Female individuals expressed in red spots (grouped by the red ellipse) and male individuals in blue spots (grouped by the blue ellipse).

Principal component analysis (PCA) showed that the first (PC 1) and second component (PC 2) were able to explain 25.2 % and 22.3 % of the variability of the results, respectively (Figure 2). In addition, it was possible to identify the formation of two separate groups along the PC 2 component. The results of females presented a greater variability and were distributed throughout the PC 1 component, while males were distributed along the PC 2 component. Notably, males are associated with Cr, V, Pb, Mo and Cd, while female individuals are associated with Hg, Mn, Cu, As and Zn. The concentration of Ba and Ni in adult individuals were lower than the LD, for this reason, they were not included in the PCA analysis.

The Kruskal-Wallis test indicated a significant difference (p = 0.01) between the concentrations of Cr in adult females (mean value < LD, n = 14) and adult males (mean value = 0.1 mg kg^-1^ d.w., n = 8). For the other trace metals, the Kruskal-Wallis test did not indicate a significant difference for the accumulation in relation to the sex of adult *Larus dominicanus*. The mean concentration of Cu in juvenile male individuals (mean value = 13 mg kg^-1^ d.w., n = 6) are higher (p = 0.04) than the concentration measured in juvenile female individuals (mean value = 9 mg kg^-1^ d.w., n = 2). The Cd concentrations in juvenile male individuals (mean value = 0.1 mg kg^-1^ d.w., n = 6) show significantly lower values (p = 0.006) than adult male individuals (mean value = 0.4 mg kg^-1^ d.w., n = 8). No significant difference was observed between female juvenile and female adult individuals.

The Kruskal-Wallis test indicated significant differences for some elements between specimens considering sex and age. However, the statistical analysis has not a good robustness due to low sample numbers, especially considering the comparison between female juveniles (n = 2) and male juveniles (n = 6). Thus, these results must be presented carefully.

### 3.2 Geographical variability

**Figure 3:**
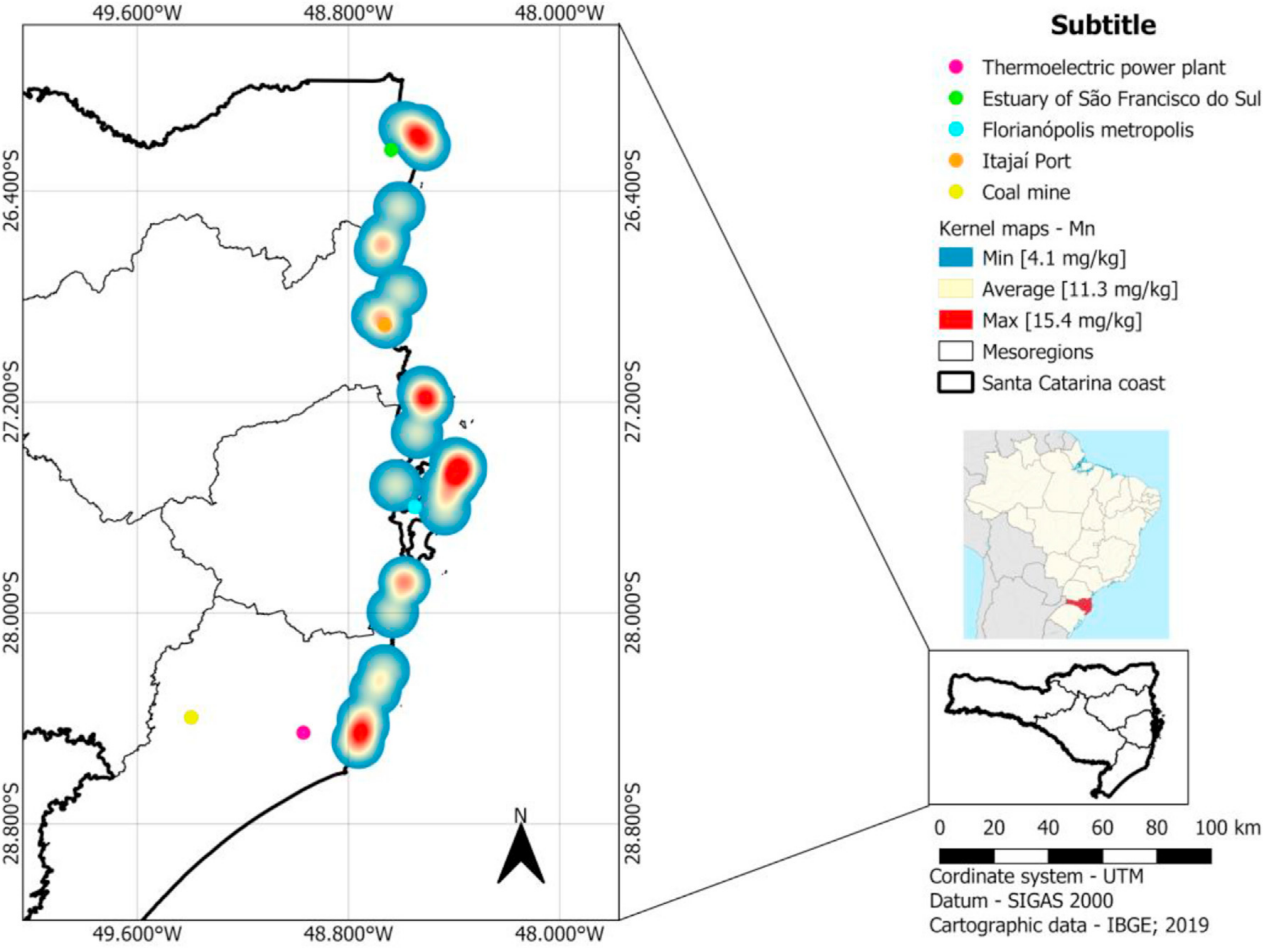
Kernel maps of Mn concentration in liver of *Larus dominicanus*.

**Figure 4:**
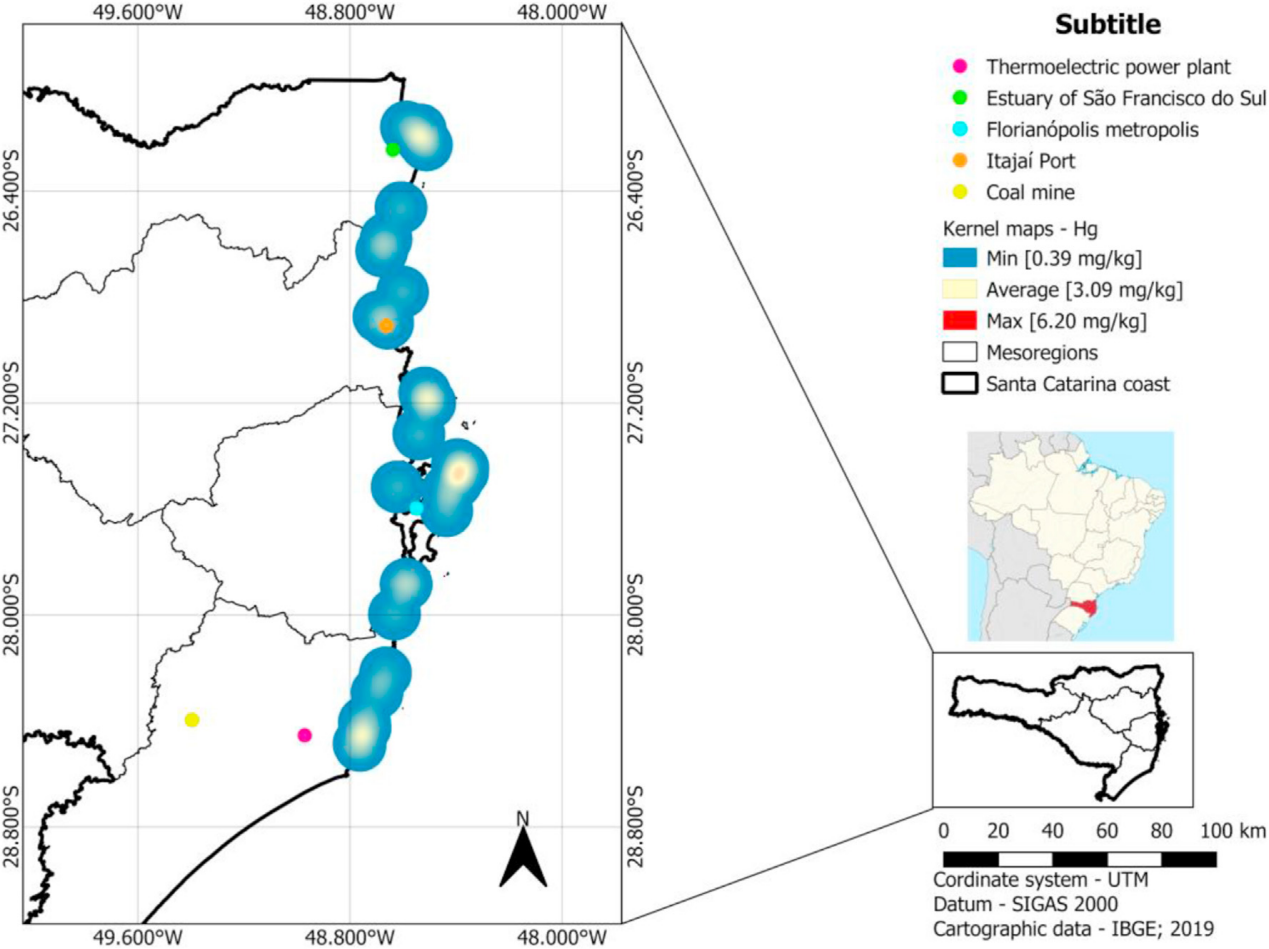
Kernel maps of Hg concentration in liver of *Larus dominicanus*.

The heat maps to trace metals Mn (Figure 3), As (Supplementary Fig. 5), Cd (Supplementary Fig. 6), Cr (Supplementary Fig. 7), Cu (Supplementary Fig. 8), Pb (Supplementary Fig. 9), Mo (Supplementary Fig. 10), Zn (Supplementary Fig. 11) and V (Supplementary Fig. 12) showed hot spots in the metropolitan region of Florianópolis (high population density), near Itajaí port, in the south of Santa Catarina state (region with several coal mines and thermoelectric power plants) and in the north of Santa Catarina state (region with several industry activities and Linguado channel). The Hg concentrations measured in liver of *Larus dominicanus* do not show any hot spots related to regions in this work (Figure 4).

The mean concentration of Cu (17 mg kg^-1^ d.w.) in individuals found in the south of Santa Catarina were significantly higher (p = 0.02, n = 5) than in the other regions. For individuals found in the metropolitan region of Florianópolis, the mean concentration of Pb (2.2 mg kg^-1^ d.w.) was significantly higher (p = 0.04, n = 8) than the mean concentration of Pb measured in individuals from other regions of Santa Catarina state. The highest concentrations of Mo (2.3 mg kg^-1^ d.w.) and Zn (217 mg kg^-1^ d.w.) were found in regions of Itajaí port, which concentrations were significantly higher (p = 0.04, n = 3) compared to other regions.

## 4 Discussion

The mean concentrations of Cu (17.6 ± 0.2 mg kg^-1^ d.w.), Hg (2.8 ± 0.3 mg kg^-1^ d.w.) and As (3.2 ± 0.4 mg kg^-1^ d.w.) reported by Moura et al. (2018) to livers of *Larus dominicanus* found dead in the coast of the state of Rio de Janeiro from Brazil were in similar ranges than concentrations of the present work. On the other hand, Cr (2.7 ± 0.4 mg kg^-1^ d.w.), Zn (253 ± 0.2 mg kg^-1^ d.w.), and Cd (1.3 ± 0.5 mg kg^-1^ d.w.) concentrations reported by Moura et al. (2018) showed higher ranges than in the present work. Cortés and Luna-Jorquera 2011 reported higher mean concentrations of Cd (9.7 ± 0.1 mg kg^-1^ d.w.) of specimens sampled in Chile than of specimens in the present study. In contrast, the mean concentrations of Cu (15.6 ± 0.08 mg kg^-1^ d.w.) were in similar ranges than Cu concentrations reported in the present work. The concentrations of Cd (0.2–0.4 mg kg^-1^ d.w.), Zn (41–44 mg kg^-1^ d.w.) and Hg (0.05–0.2 mg kg^-1^ d.w.) found by Numata et al. (2008) and Cd (0.1 mg kg^-1^ d.w.), Zn (25 mg kg^-1^ d.w.) and Hg (0.4 mg kg^-1^ d.w.) Lock et al. (1992) in individuals sampled in New Zealand were in lower ranges than the values found in individuals collected in South America (Table 3). The concentrations of trace elements found in livers of dead *Larus dominicanus* on the coast of Santa Catarina state were in similar ranges than those found in other regions of Brazil and in other countries (Table 3) (Moura et al., 2018; Vermeer and Castilla 1991; Numata et al., 2008; Cortés and Luna-Jorquera 2011; Lock et al., 1992). This indicates that specimen of *Larus dominicanus* from Santa Catarina state are not exposed to explicitly higher contamination risks than specimens from the other investigated regions of the world.

**Table 3:** Average concentrations of trace metals determined in liver *of Larus dominicanus* in the world.

Author	Country	As	Cd	Cr	Cu	Pb	Mn	Mo	Zn	Ni	Ba	V	Se	Hg
**This study**	**SC**	**2.7**	**0.4**	**<LD**	**14**	**0.63**	**11**	**1.9**	**133**	**<LD**	**<LD**	**0.4**	**-**	**3.1**
MOURA et al.	RJ	3.2	1.3	2.7	17.6	-	-	-	253	-	-	-	5.9	2.8
VERMEER and CASTILLA	CH	-	0.9	-	2.1	-	-	-	-	-	-	-	-	-
CORTÉS and LUNA-JORQUERA	CH	-	9.7	-	15.6	-	-	-	-	-	-	-	-	-
*NUMATA* et al.	NZ	<LD	0.2–0.4	-	28–76	0.02–0.11	-	-	41–44	-	-	-	-	0.05–0.2
LOCK et al.	NZ	-	0.1	-	7.5	25.80	-	-	25	-	-	-	-	0.4

SC = Santa Catarina state - Brazil; RJ = Rio de Janeiro state - Brazil; CH = Chile; NZ = New Zealand. All results are expressed in (mg kg^−1^ d.w.)

The differences between the accumulations of trace metals in *Larus dominicanus* from different parts of the world may be explained by their opportunistic feeding habit, which makes this species less selective in relation to the choice of its prey. Silva-Costa and Bugoni (2013) observed that the diet of *Larus dominicanus* living in South of Brazil consists of fish, bivalves and molluscs as well as of waste, leftover food and bones. These food sources contribute to 71.2 %, 14.9 % and 1.9 % of the food habit of *Larus dominicanus*, respectively (Silva-Costa and Bugoni 2013). Furthermore, Lock et al. (1992) presumed that *Larus dominicanus* inhabiting New Zealand have generalistic and opportunistic habits, being able to feed on the remains of other animals frequently. Marón et al. (2015) reported that *Larus dominicanus* of the peninsula of Valdés in Argentina feed of fat and skin of breeding or dead whales. Basically, the fact that *Larus dominicanus* is not selective in relation to its feeding habits can explain the expressive accumulation of nonessential elements as As, Pb, Cd, Hg, V and Ba.

For the first time, the present study presented lower concentrations of trace elements in livers of female adult individuals than female juvenile individuals of *Larus dominicanus* living in Brazil. This can be explained as female adults transfer metals to eggs during reproduction (Monteiro and Furness 1955; Becker 1992). Also moulting feathers can be an important mechanism of detoxification of Hg, Sb, Ag, Cu or Pb, which indicated the higher concentrations of metals in juvenile individuals that had not yet undergone moulting of feathers (Honda et al., 1985; Jakimska et al., 2011).

Homeostatic interactions can control the accumulation of trace elements, especially of Cd. Thus, the presence of this metal in the liver of birds may indicate long-term exposure (Jakimska et al., 2011). In concordance with the results of reported by Cortés and Luna-Jorquera (2011) for livers of *Larus dominicanus* in Chile, results of the present work indicate higher concentrations of Cd in adult individuals when compared with juvenile individuals. This difference can be associated with the chronic exposure to Cd. Metabolic processes from the translocation of mainly Zn from metallothionine cause greater accumulations of Cd in the liver, which indicates processes of bioaccumulation in adult individuals (Jakimska et al., 2011a; 2011b). In this way, the results showed in the present study indicate the existence of an important mechanism of bioaccumulation of Cd related to the age of *Larus dominicanus*.

Metals such as Cu, Zn and Mn are essential for the metabolism or formation of pigments in bird feathers. Thus, these elements can indicate an important role in the development of young individuals, especially for the initial growth of feathers (Ribeiro et al., 2009; Niecke et al., 1999). Significant differences were observed for the concentrations of Cu in juvenile and adult individuals found on the coast of Santa Catarina state. These results were similar with those obtained by Cortés and Luna-Jorquera (2011), suggesting that juvenile individuals metabolize higher concentrations of Cu, possibly destined to the growth and pigmentation of the feathers. Just a few studies report the accumulation of Cr in seabirds. Ribeiro et al. (2009) suggested that high concentrations of Cr may express levels of contamination of the breeding area of seabirds. In this way, the results of the present work can suggest that the bioaccumulation of Cr in male individuals may be influenced by the foraging area.

The Hg translocation in birds has been reported by models that consider dietary intake, absorption in the intestine, transport in the blood, accumulation in tissues such as liver, kidney and muscles, and excretion through feathers (Monteiro and Furness 1955). In the study of Honda et al. (1985), the authors suggested that feathers contain 70 % of the total Hg concentration present in the organism of several species of seabirds and land birds. The elimination of Hg through the feathers was experimentally tested by Lewis and Furness (1991) in *Larus ridibandus* individuals. Here, a progressive increase in the concentration of Hg in feathers was observed in relation to feather growth. Considering that bird plumage is renewed annually, generally after breeding, geographically related results obtained in the present study suggest that the concentration of Hg measured in *Larus dominicanus* collected along the coast of Santa Catarina is invariable in relation to the habitat. It indicates that Hg is being regulated by a mechanism of detoxification, possibly related to the moulting of feathers. However, this hypothesis needs to be evaluated through increased a number of samples analyzed and simultaneous measurements of Hg in feathers and livers of individuals living on the coast of Santa Catarina.

The evaluation of the accumulation of trace metals in marine animals is important for the understanding of coastal ecosystems (Moller 1996). Port regions contribute to a large number of contaminants which can be bioavailable to the food chain (Caro et al., 2015). The results obtained in the present work presume through Kernel density maps that the bioaccumulation of metals is associated to the habitat, corroborating with the results obtained by Cortés and Luna-Jorquera (2011) and Numata et al. (2008). A hotspot of the Kernel map represents a range of about 11 km. Thus, it expresses with a very high probability in which range the sampled specimen died as only fresh dead specimen were collected. It is improbable that a sampled specimen died out of this range and was in appropriate sample conditions on the sampling point.

The higher bioaccumulation of Cu for individuals collected in the South of Santa Catarina region may be associated with the fact that the port of Imbituba city is an important route of mineral coal transport, which may is contributing to the increase of the bioavailability of Cu for the individuals that inhabit this region (Gomes et al., 2003). In addition, the use of antifouling paints is a critical source of Cu and Zn in the trophic chain of marine animals, more specifically for birds, which can bioaccumulate this metal through the consumption of fish and molluscs (Honda et al., 1985; Srinivasan and Swain 2007).

The metropolitan region of Florianópolis is the region with the highest urban density in the state and has urban beaches. This may have contributed to the accumulation of Pb in individuals of *Larus dominicanus* collected in the metropolitan region of Florianópolis. The Itajaí region has great economic importance for the state of Santa Catarina with emphasis on the textile industry and the region possess the largest port in the state (Itajaí port) (Pereira Filho et al., 2003). The antifouling paints used in the ships in this region may have contributed to the accumulation of Mo and Zn in specimens of *Larus dominicanus* collected in the region of Itajaí.

## 5 Conclusions

The results presented in this work showed distinct intraspecific variations of trace elements in hepatic tissues of *L. dominicanus* specimens and provides the first background levels for hepatic tissue of the species for the coast region of Santa Catarina State. In general, concentrations of trace elements found in livers of *Larus dominicanus* were comparable with values reported in other studies in South America and other regions of the world. This shows that possibly individuals living on the southern coast of Brazil are subject to the same levels of contamination as individuals living in other places of the world. Conducting bioaccumulation studies of trace elements in bird species like *Larus dominicanus*, which inhabit different parts of the world, are essential to establish levels of contamination of coastal environments.

The Kernel density maps showed to be a promising tool to identify influences of the locality on bioaccumulation of trace elements in *Larus dominicanus*. Besides feeding habit and habitat, the individual age showed to be an influence factor of intraspecific differences in bioaccumulation, especially for Cd.

## Declarations

### Author contribution statement

J.H. Pedrobom and A.A. Menegário: Conceived and designed the experiments; Performed the experiments; Analyzed and interpreted the data; Contributed reagents, materials, analysis tools or data; Wrote the paper.

H. Gemeiner, E.T. Sulato and L.P. Elias: Performed the experiments; Analyzed and interpreted the data; Contributed reagents, materials, analysis tools or data; Wrote the paper.

P.P. Serafini, C.J. Rodrigues, A.S. Barreto and M.A.G. de Araújo Júnior: Analyzed and interpreted the data; Wrote the paper.

### Funding statement

This research did not receive any specific grant from funding agencies in the public, commercial, or not-for-profit sectors.

### Data availability statement

Data included in article/supplementary material/referenced in article.

### Declaration of interests statement

The authors declare no conflict of interest.

### Additional information

No additional information is available for this paper.

## Appendix A Supplementary data

Suplementary Data R4 Suplementary Data R4
