# Supplementary material for: Intraspecific variation of trace elements in the kelp gull (Larus dominicanus): influence of age, sex and location
Source: Heliyon. 2021 Jan 18;7(1):e05994. doi: 10.1016/j.heliyon.2021.e05994 (PMC7820554; doi:10.1016/j.heliyon.2021.e05994)
Supplement: Suplementary Data R4 [file mmc1.docx]

**SUPPLEMENTARY DATA**

**Intraspecific variation of trace elements in the kelp gull (*Larus Dominicanus)*: INFLUENCE OF AGE, SEX AND LOCATION**

Jorge Henrique Pedrobom^a^, Amauri A. Menegário^a*^, Hendryk Gemeiner^a^, Everton Tiago Sulato^a^, Lucas Pellegrini Elias^a^, Patrícia Pereira Serafini^b^, Claudinei José Rodrigues^b^, André S. Barreto^c^, Marcus Antônio Gonçalves de Araújo Júnior^d^,

^a^ Environmental Studies Center (CEA), São Paulo State University (UNESP), Avenida 24-A, 1515, CEP 13506-900, Rio Claro, SP, Brazil.

^b^ Chico Mendes Institute for Biodiversity Conservation – ICMBio, Rodovia Jornalista Maurício Sirotski Sobrinho, km 2, CEP 88053-700, Florianópolis, SC, Brazil.

^c^ Biodiversity and Geoprocessing Informatics Laboratory, University of Vale do Itajaí (UNIVALI), Rua Uruguai, 458, CEP 88302-901, Itajaí, SC, Brazil.

^d^ Research and Development Center Leopoldo Américo Miguez de Mello – CENPES, PETROBRAS - Petróleo Brasileiro S.A, Avenida Horácio Macedo, 950, CEP 21941-915, Rio de Janeiro, RJ, Brazil.

* Corresponding author.

Tel./fax.: +55-1935269491

Environmental Studies Center (CEA), São Paulo State University (UNESP), Avenida 24-A, 1515, CEP 13506-900, Rio Claro, SP, Brazil.

E-mail address: amauri.antonio-menegario@unesp.br

**Author Contributions Statement**

Conceived and designed the experiments; performed the experiments; analyzed and interpreted the data; contributed reagents, materials, analysis tools or data; wrote the paper: Jorge Henrique Pedrobom, Amauri A. Menegário

Performed the experiments; analyzed and interpreted the data; contributed reagents, materials, analysis tools or data and wrote the paper: Hendryk Gemeiner, Everton Tiago Sulato, Lucas Pellegrini Elias

Analyzed and interpreted the data and wrote the paper: Patrícia Pereira Serafini, Claudinei José Rodrigues, André S. Barreto, Marcus Antônio Gonçalves de Araújo Júnior





**Supplementary Fig. 5** Kernel maps of As concentration in liver of *Larus dominicanus*.





**Supplementary Fig. 6** Kernel maps of Cd concentration in liver of *Larus dominicanus*.





**Supplementary Fig. 7** Kernel maps of Cr concentration in liver of *Larus. dominicanus*.





**Supplementary Fig. 8** Kernel maps of Cu concentration in liver of *Larus dominicanus*.





**Supplementary Fig. 9** Kernel maps of Pb concentration in liver of *Larus dominicanus*.





**Supplementary Fig. 10** Kernel maps of Mo concentration in liver of *Larus dominicanus*.





**Supplementary Fig. 11** Kernel maps of Zn concentration in liver of *Larus dominicanus*.





**Supplementary Fig. 12** Kernel maps of V concentration in liver of *Larus dominicanus*

| **Supplementary Tab. 3** General data of samples. Concentration of trace elements in mg kg^-1^ d.w. | | | | | | | | | | | | | | | | | |
| --- | --- | --- | --- | --- | --- | --- | --- | --- | --- | --- | --- | --- | --- | --- | --- | --- | --- |
| **Sample colection** | **Local (City)** | **Sex** | **Age** | **Latitude** | **Longitude** | **As** | **Cd** | **Cr** | **Cu** | **Pb** | **Mn** | **Mo** | **Zn** | **Ni** | **Ba** | **V** | **Hg** |
| 10/31/2016 | Laguna | Male | Juvenile | -28.4853 | -48.76555 | 0.82 | 0.07 | < LD | 13.17 | 0.05 | 10.70 | 1.86 | 303.26 | < LD | 0.11 | 0.36 | 0.90 |
| 11/03/2016 | Imbituba | Male | Juvenile | -28.29325 | -48.69813 | 1.90 | 0.06 | < LD | 9.48 | 0.03 | 9.50 | 1.42 | 70.64 | < LD | < LD | 0.21 | 1.80 |
| 03/23/2017 | Bombinhas | Male | Juvenile | -27.1909535 | -48.505735 | 1.58 | 0.27 | < LD | 14.51 | 0.14 | 11.43 | 2.40 | 91.00 | < LD | < LD | 0.55 | 3.22 |
| 03/08/2018 | Penha | Male | Juvenile | -26.781608 | -48.6019473 | 6.98 | 0.10 | < LD | 17.30 | 0.03 | 14.21 | 1.36 | 296.33 | < LD | < LD | 0.46 | 6.25 |
| 02/20/2018 | Itapoá | Male | Juvenile | -26.1577286 | -48.5871518 | 3.17 | 0.15 | < LD | 13.31 | 0.03 | 6.35 | 1.98 | 222.21 | < LD | 0.20 | 0.31 | 5.50 |
| 03/16/2018 | Florianópolis | Male | Juvenile | -27.5164232 | -48.6219517 | 1.12 | 0.09 | < LD | 12.01 | 0.06 | 9.52 | 1.75 | 78.19 | < LD | < LD | 0.10 | 4.17 |
| 05/04/2018 | Araquari | Male | Adult | -26.5801549 | -48.6637893 | 0.38 | 0.21 | < LD | 13.68 | 0.04 | 14.54 | 2.27 | 115.89 | 0.11 | < LD | 0.45 | 1.43 |
| 05/03/2018 | Florianópolis | Male | Adult | -27.4602037 | -48.3754266 | 1.38 | 0.25 | < LD | 12.66 | 0.14 | 10.91 | 1.86 | 77.70 | < LD | < LD | 0.11 | 1.27 |
| 06/08/2017 | Governador Celso Ramos | Male | Adult | -27.3169244 | -48.5401522 | 4.94 | 0.40 | < LD | 13.85 | 0.20 | 7.62 | 1.97 | 73.10 | < LD | < LD | 0.27 | 0.39 |
| 05/31/2017 | Florianópolis | Male | Adult | -27.49815 | -48.39937 | 1.10 | 0.28 | 0.15 | 11.26 | 0.47 | 10.80 | 1.70 | 74.84 | < LD | < LD | 4.62 | 2.57 |
| 11/16/2017 | Balneário Barra Do Sul | Male | Adult | -26.4625656 | -48.6076297 | 1.95 | 0.48 | 0.29 | 17.61 | 0.11 | 11.45 | 2.72 | 80.04 | < LD | < LD | 0.28 | 4.35 |
| 10/17/2017 | Florianópolis | Male | Adult | -27.52826 | -48.43015 | 2.13 | 0.20 | 0.15 | 11.77 | 0.04 | 10.73 | 1.67 | 110.12 | < LD | < LD | 0.13 | 5.78 |
| 12/08/2017 | Itajaí | Male | Adult | -26.8738193 | -48.6905675 | 1.39 | 0.52 | < LD | 11.87 | 0.04 | 15.07 | 2.08 | 300.00 | < LD | < LD | 0.05 | 3.73 |
|  |  |  |  |  |  |  |  |  |  |  |  |  |  |  |  |  | Continue |
| **Supplementary Continuation Tab. 3** General data of samples. Concentration of trace elements is mg kg^-1^ d.w. | | | | | | | | | | | | | | | | | |
| **Sample colection** | **Local (City)** | **Sex** | **Age** | **Latitude** | **Longitude** | **As** | **Cd** | **Cr** | **Cu** | **Pb** | **Mn** | **Mo** | **Zn** | **Ni** | **Ba** | **V** | **Hg** |
| 03/01/2018 | São Francisco Do Sul | Male | Adult | -26.2094017 | -48.525397 | 4.34 | 0.82 | < LD | 15.77 | 0.02 | 12.00 | 1.71 | 81.34 | < LD | < LD | 0.33 | 1.54 |
| 05/03/2017 | São Francisco Do Sul | Female | Juvenile | -26.2253298 | -48.5119616 | 6.69 | 0.22 | < LD | 9.23 | 0.08 | 13.72 | 2.14 | 383.69 | < LD | < LD | 0.47 | 4.69 |
| 11/21/2017 | Palhoça | Female | Juvenile | -27.8839029 | -48.5883772 | 5.02 | 0.10 | < LD | 8.84 | 0.04 | 11.72 | 1.43 | 125.19 | < LD | < LD | 0.01 | 4.89 |
| 01/02/2018 | Imbituba | Female | Adult | -28.2183675 | -48.6661935 | 1.99 | 0.62 | < LD | 25.77 | 0.04 | 15.23 | 1.91 | 210.03 | < LD | < LD | 0.06 | 2.87 |
| 05/16/2018 | Bombinhas | Female | Adult | -27.1993509 | -48.4993254 | 2.32 | 0.11 | < LD | 14.21 | 0.18 | 12.54 | 2.13 | 224.96 | < LD | < LD | 0.16 | 2.91 |
| 05/13/2018 | Laguna | Female | Adult | -28.4037828 | -48.7455225 | 1.69 | 1.20 | < LD | 18.00 | 0.02 | 14.44 | 2.82 | 108.56 | < LD | 0.11 | 0.27 | 3.60 |
| 05/29/2018 | Laguna | Female | Adult | -28.492079 | -48.762498 | 0.63 | 0.68 | < LD | 12.91 | 0.11 | 10.11 | 2.52 | 105.36 | < LD | < LD | 0.30 | 4.39 |
| 11/27/2017 | Florianópolis | Female | Adult | -27.4319072 | -48.3947609 | 1.61 | 0.25 | < LD | 11.62 | 0.16 | 8.43 | 1.64 | 118.37 | < LD | < LD | 0.08 | 1.22 |
| 04/01/2018 | Florianópolis | Female | Adult | -27.4441154 | -48.374341 | 0.17 | 0.80 | < LD | 11.31 | 16.18 | 8.33 | 1.86 | 84.21 | < LD | < LD | 0.12 | 0.84 |
| 05/10/2017 | Bombinhas | Female | Adult | -27.1615049 | -48.518339 | 1.44 | 0.32 | < LD | 15.75 | 0.05 | 12.58 | 2.68 | 125.16 | < LD | < LD | 0.30 | 2.78 |
| 05/22/2017 | São Francisco Do Sul | Female | Adult | -26.1681736 | -48.5347053 | 11.70 | 1.10 | < LD | 15.16 | 0.03 | 10.48 | 2.10 | 105.60 | < LD | < LD | 0.22 | 6.08 |
| 04/26/2017 | Florianópolis | Female | Adult | -27.6061 | -48.43499 | 0.46 | 0.49 | < LD | 8.67 | 0.24 | 11.87 | 1.50 | 67.28 | < LD | 0.10 | 0.05 | 1.43 |
| 05/13/2017 | Garopaba | Female | Adult | -27.99619 | -48.6321 | 1.75 | 0.37 | < LD | 14.27 | 0.03 | 11.06 | 1.99 | 89.90 | < LD | < LD | 0.44 | 1.05 |
| Continue | | | | | | | | | | | | | | | | | |
| **Supplementary Continuation Tab. 3**  General data of samples. Concentration of trace elements is mg kg^-1^ d.w. | | | | | | | | | | | | | | | | | |
| **Sample colection** | **Local (City)** | **Sex** | **Age** | **Latitude** | **Longitude** | **As** | **Cd** | **Cr** | **Cu** | **Pb** | **Mn** | **Mo** | **Zn** | **Ni** | **Ba** | **V** | **Hg** |
| 09/29/2017 | Laguna | Female | Adult | -28.4248042 | -48.7432579 | 0.56 | 0.38 | < LD | 15.52 | 0.09 | 4.06 | 0.92 | 35.87 | < LD | < LD | 0.15 | 1.08 |
| 09/30/2017 | Palhoça | Female | Adult | -27.8844773 | -48.5881781 | 8.33 | 0.67 | < LD | 14.68 | 0.05 | 13.07 | 1.86 | 67.61 | < LD | < LD | 0.10 | 4.56 |
| 09/12/2017 | Barra Velha | Female | Adult | -26.6264527 | -48.6802436 | 4.09 | 0.42 | < LD | 14.46 | 0.01 | 15.43 | 2.20 | 84.98 | < LD | < LD | 0.01 | 5.76 |
| 01/03/2018 | Navegantes | Female | Adult | -26.8983053 | -48.658061 | 0.34 | 0.18 | < LD | 14.44 | 0.07 | 10.90 | 2.04 | 72.88 | < LD | < LD | 0.03 | 1.55 |

| **Supplementary Tab. 4** Points of the scatter plot of Principal Components Analysis. | | | |
| --- | --- | --- | --- |
|  | **PC 1** | **PC 2** | **PC 3** |
| **Eigenvalue** | 2.6 | 2.3 | 1.4 |
| **Variance (%)** | 25.2 | 22.3 | 13.1 |
| **Scatter plot - PCA** | | | |
| **Male** | 0.76 | 0.26 | -1.89 |
|  | -0.64 | 0.66 | -0.52 |
|  | -0.69 | 0.97 | 1.59 |
|  | -1.44 | 4.56 | -0.11 |
|  | 3.36 | 3.13 | -0.40 |
|  | 0.71 | 1.16 | -0.35 |
|  | 1.70 | -0.11 | -1.10 |
|  | 1.27 | 0.11 | 1.87 |
| **Female** | 1.34 | -1.75 | -0.48 |
|  | -0.23 | -1.19 | -1.27 |
|  | 1.80 | -0.53 | 0.28 |
|  | 0.10 | -0.32 | -0.14 |
|  | -1.91 | -0.99 | -0.35 |
|  | -2.77 | 1.24 | 0.81 |
|  | 0.24 | -0.58 | -1.06 |
|  | 1.46 | -0.93 | 3.02 |
|  | -1.63 | -0.96 | -0.45 |
|  | -1.15 | -0.12 | -0.44 |
|  | -3.06 | -0.66 | 0.83 |
|  | 0.67 | -1.26 | 1.42 |
|  | 1.17 | -1.53 | -0.26 |
|  | -1.07 | -1.15 | -1.00 |

| **Supplementary Tab. 4** Points of the vector variables of Principal Components Analysis | | | |
| --- | --- | --- | --- |
|  | **PC 1** | **PC 2** | **PC 3** |
| **As** | 0.21 | -0.10 | 0.65 |
| **Cd** | 0.30 | 0.08 | 0.54 |
| **Cr** | 0.34 | 0.76 | -0.12 |
| **Cu** | 0.32 | -0.10 | 0.09 |
| **Pb** | -0.31 | 0.46 | 0.14 |
| **Mn** | 0.44 | -0.11 | -0.36 |
| **Mo** | 0.42 | 0.07 | -0.22 |
| **Zn** | 0.14 | -0.14 | -0.23 |
| **V** | -0.08 | 0.38 | 0.02 |
| **Hg** | 0.40 | -0.05 | 0.13 |
